# Cost-effective prognostic evaluation of breast cancer: using a STAR nomogram model based on routine blood tests

**DOI:** 10.3389/fendo.2024.1324617

**Published:** 2024-03-11

**Authors:** Caibiao Wei, Yihua Liang, Dan Mo, Qiumei Lin, Zhimin Liu, Meiqin Li, Yuling Qin, Min Fang

**Affiliations:** ^1^ Department of Clinical Laboratory, Guangxi Medical University Cancer Hospital, Nanning, Guangxi, China; ^2^ Department of Breast, Guangxi Zhuang Autonomous Region Maternal and Child Health Care Hospital, Nanning, China; ^3^ Guangxi Clinical Research Center for Anesthesiology, Guangxi Medical University Cancer Hospital, Nanning, Guangxi, China

**Keywords:** breast cancer, prognosis, nomogram, survival, routine blood tests, C-index

## Abstract

**Background:**

Breast cancer (BC) is the most common and prominent deadly disease among women. Predicting BC survival mainly relies on TNM staging, molecular profiling and imaging, hampered by subjectivity and expenses. This study aimed to establish an economical and reliable model using the most common preoperative routine blood tests (RT) data for survival and surveillance strategy management.

**Methods:**

We examined 2863 BC patients, dividing them into training and validation cohorts (7:3). We collected demographic features, pathomics characteristics and preoperative 24-item RT data. BC risk factors were identified through Cox regression, and a predictive nomogram was established. Its performance was assessed using C-index, area under curves (AUC), calibration curve and decision curve analysis. Kaplan-Meier curves stratified patients into different risk groups. We further compared the STAR model (utilizing HE and RT methodologies) with alternative nomograms grounded in molecular profiling (employing second-generation short-read sequencing methodologies) and imaging (utilizing PET-CT methodologies).

**Results:**

The STAR nomogram, incorporating subtype, TNM stage, age and preoperative RT data (LYM, LYM%, EOSO%, RDW-SD, P-LCR), achieved a C-index of 0.828 in the training cohort and impressive AUCs (0.847, 0.823 and 0.780) for 3-, 5- and 7-year OS rates, outperforming other nomograms. The validation cohort showed similar impressive results. The nomogram calculates a patient’s total score by assigning values to each risk factor, higher scores indicating a poor prognosis. STAR promises potential cost savings by enabling less intensive surveillance in around 90% of BC patients. Compared to nomograms based on molecular profiling and imaging, STAR presents a more cost-effective, with potential savings of approximately $700-800 per breast cancer patient.

**Conclusion:**

Combining appropriate RT parameters, STAR nomogram could help in the detection of patient anemia, coagulation function, inflammation and immune status. Practical implementation of the STAR nomogram in a clinical setting is feasible, and its potential clinical impact lies in its ability to provide an early, economical and reliable tool for survival prediction and surveillance strategy management. However, our model still has limitations and requires external data validation. In subsequent studies, we plan to mitigate the potential impact on model robustness by further updating and adjusting the data and model.

## 1 Introduction

Breast cancer (BC) stands as the most common malignancy in women, and its incidence continues to rise at an alarming rate (1, 2). This escalating trend highlights the urgent need for improved strategies in both prevention and treatment. A significant challenge in addressing BC lies in its inherent heterogeneity, where patients with the same stage and treatment can exhibit markedly different outcomes (3). Tumor staging, hormone receptor status, HER2 expression, molecular subtypes, and genomic alterations are all potential contributors to the heterogeneity of breast cancer (4). Given this prognostic heterogeneity, it is crucial to enhance prognostic stratification and treatment outcome prediction in order to develop individualized treatment regimens. Precisely determining the prognosis at the time of diagnosis is essential to avoid overtreatment of nonaggressive cases and undertreatment of aggressive forms of the disease (5).

Prognostic models for BC patients have yielded significant achievements, encompassing key factors like pathological features, molecular profiling, and imaging characteristics (6–10). However, these models are not without their limitations. For example, molecular profiling based on gene expression profiles may be influenced by systemic factors such as chronic or transient inflammatory diseases or other non-cancerous diseases (11). The interpretation of imaging models introduces subjectivity due to variations in experience, professional knowledge, and personal bias among physicians. Consequently, different healthcare professionals may yield diverse diagnostic results when analyzing the same set of images (12, 13). Furthermore, the utilization of advanced imaging techniques such as PET-PET/CT and whole-body MRI comes with a substantial cost and potential radiation risks, leading to adverse effects on intensive follow-up programs and causing psychological distress (14). Although these models successfully predict the outcome of patients, the acquisition of these features is invasive and expensive, so these tools are difficult to promote. As a result, the widespread adoption and applicability of these models in routine clinical practice have been hindered. To address these gaps in current breast cancer prognostic models, it is imperative to explore affordable and reliable prognostic factors to improve the precision and feasibility of these models.

The routine blood test (RT) is a straightforward, quick, and the most common examination performed on nearly all cancer patients upon admission; it gives vital information regarding human metabolism, inflammation, and internal environmental factors (15, 16). In addition to the function of assisting in the diagnosis of disease, RT examination is also the most commonly used indicator to observe the treatment effect, medication or withdrawal, continued or stopped treatment, disease recurrence or recovery (17–19). There is new evidence that RT parameters may provide prognostic information for cancer patients. Some hematological markers, such as inflammatory index and red blood cell distribution width, have obvious advantages in predicting prognosis (20–22). Inflammation plays a crucial regulatory role in the development of breast cancer (23), and hematological biomarkers can provide valuable clues about pathophysiological changes (24, 25). Research has shown that a higher lymphocyte proportion is positively correlated with a favorable prognosis (26), whereas a lower percentage of eosinophils (27), higher red blood cell distribution width (28), and higher platelet large cell ratio (29) are associated with a poorer prognosis. Therefore, an in-depth examination of the association between preoperative RT indicators and the prognosis of BC patients offers a good reference for physicians, which has considerable clinical value and practical possibilities.

To address these gaps in current breast cancer prognostic models, our research focuses on exploring the relationship between preoperative blood routine indicators and prognosis in breast cancer patients, and on developing and validating the STAR nomogram as an accurate method for predicting the prognosis of breast cancer patients. Our research, to the best of our knowledge, represents the first systematic effort to develop and rigorously validate a nomogram based on comprehensive preoperative RT indicators for accurately predicting BC patient prognosis. Consequently, a comprehensive investigation into the association between preoperative RT indicators and the prognosis of BC patients was conducted. We simultaneously collected data on 24 pre-treatment RT indicators from a substantial cohort of nearly 3,000 BC-diagnosed patients. Compared to traditional predictive models, the STAR line chart utilizes non-invasive and cost-effective blood tests, providing a simpler and more practical forecasting tool.

## 2 Materials and methods

### 2.1 Patients

During the period spanning from July 2013 to December 2021, we meticulously gathered data from a total of 4018 cases of BC patients diagnosed at the tumor hospital of Guangxi Medical University. Our study included 2,863 BC patients who met the inclusion criteria and were selected from this large cohort. However, 1155 cases did not fulfill the specified criteria and were subsequently excluded from the analysis.

The inclusion criteria were: (I) Patients that were confirmed by pathological examination diagnosed with malignant breast cancer; (II) complete clinical and routine blood test information was available for all patients; (III) patients consented to post-operative follow-up visits. The exclusion criteria were: (I) Patients with non-primary breast cancer: We have excluded patients with non-primary breast cancer to maintain the specificity of our study. The prognosis of primary breast cancer may differ from other breast cancer types. Therefore, we focused on patients with primary breast cancer to minimize potential prognostic heterogeneity. (II) Patients with ductal carcinoma *in situ* (DCIS): DCIS is a precancerous lesion that typically has a favorable prognosis in the early stages. Our prognostic evaluation focuses primarily on breast cancer types with potential for malignancy. (III) Patients with concurrent primary tumors: We excluded patients with additional primary tumors to ensure our study solely encompasses the prognostic evaluation of breast cancer, avoiding interference from other tumor types. (IV) Patients who received any preoperative anti-tumor therapy: We excluded patients who received any preoperative anti-tumor therapy to minimize the influence of treatment on outcomes. Anti-tumor therapy may alter the levels of hematological and inflammatory biomarkers and independently impact prognosis. (V) Patients with acute or chronic inflammatory diseases: We excluded patients with acute or chronic inflammatory diseases to avoid the impact of Blood routine test results, allowing a more accurate assessment of the association between breast cancer prognosis and these markers. (VI) Patients lost to follow-up: We excluded patients lost to follow-up to ensure sufficient data for tracking prognosis outcomes. Loss to follow-up may lead to incomplete data and biased results, which could affect the reliability analysis of the association between Blood routine test results and breast cancer prognosis.

### 2.2 Ethics approval and consent to participate

Our study was approved by Guangxi Medical University Cancer Hospital Ethical Review Committee (Approve No.LW2023084) and conducted following the ethical principles outlined in the Helsinki Declaration of 1964 and its subsequent amendments or other ethical standards with equivalent requirements. To ensure patient confidentiality, the identities of the individuals included in this study were anonymized using computer-generated ID numbers. On admission, all patients provided written consent for their anonymized medical data to be analyzed and published for research purposes.

### 2.3 Data collection and classification

Data collection was by two independent investigators, CBW and YHL, with validation by a third investigator, MF. From the patients’ medical records, the demographic characteristics, clinical characteristics, and results of preoperative RT were extracted. The study collected data on various factors, including age, histologic type, grade, subtype, TNM stage, and results of routine laboratory blood test items. The RT data encompassed the following parameters: white blood cell (WBC), red blood cell (RBC), hemoglobin (HGB), platelet (PLT), lymphocyte (LYM), percentage of lymphocyte (LYM%), monocyte (MONO), percentage of monocyte (MONO%), neutrophils (NEU), percentage of neutrophils (NEU%), eosinophils (EOSO), percentage of Eosinophils (EOSO%), basophil (BASO), percentage of basophil (BASO%), hematocrit (HCT), mean corpuscular volume (MCV), mean corpuscular hemoglobin (MCH), mean corpuscular hemoglobin concentration (MCHC), red blood cell distribution width - standard deviation (RDW-SD), red blood cell distribution width - coefficient of variation (RDW-CV), mean platelet volume (MPV), platelet crit (PCT), platelet distribution width (PDW) and platelet-large cell ratio (P-LCR). The TNM staging system used in the study was based on the American Joint Committee on Cancer (AJCC) Version 8 Cancer Staging System (30). The tumor grade classification followed the guidelines of the National Comprehensive Cancer Network (NCCN) 2022 guideline clinical staging system. Utilizing receiver operating characteristic (ROC) curves, the optimal cut-off values for each numerical index were determined.

### 2.4 Follow-up

Patients in the study were subject to follow-up procedures conducted either via phone or through an outpatient surveillance system. These follow-ups aimed to gather information about the patient’s current condition or, in cases where the patient had already passed away, to obtain the date of death. The primary endpoint of the study was overall survival (OS), which was defined as the duration from the initial diagnosis to the occurrence of death. For patients who survived to the conclusion of the investigation, the date of their final follow-up was regarded as the study’s endpoint. In contrast, the date of mortality was considered the study endpoint for patients who had passed away prior to the conclusion of the study. The most recent follow-up took place in December 2022. To address potential biases or challenges encountered during follow-up, we utilize Complete Case Analysis (CCA) (31).

### 2.5 Cost-effectiveness analysis

Two authors (CBW and YHL) conducted independent searches in the PubMed and Web of Science databases utilizing the following search terms: (Breast Neoplasm OR Breast Tumor OR Breast Cancer) AND (Nomogram) AND (Prognosis). The literature was systematically reviewed to identify representing BC survival prediction models based on molecular profiling or imaging features. Literature inclusion criteria were established as follows: (I)studies not limiting any breast cancer subtype; (II) investigations exploring the association between BC survival and molecular profiling or imaging features; (III) studies representing BC survival prediction models based on molecular profiling or imaging features; (IV) availability of both a training cohort and a validation cohort with a C-index value; and (V) inclusion of a Chinese cohort. Abstracts and full-texts underwent independent screening by CBW and YHL, with any disagreements resolved through consensus with a third author (MF). Ultimately, the studies by Silei Sui et al. and Xiaojun Xu et al. were selected to represent nomograms based on molecular profiling and imaging features, respectively (32, 33). In our cost analysis, we considered the direct unit costs associated with various nomograms in China. The expenses linked to TNM staging were determined using a hematoxylin-eosin (HE) methodology, while the costs associated with molecular profiling were gathered through second-generation short-read sequencing. In contrast, the expenses related to image profiling were acquired through PET-CT. The comprehensive costs for the STAR nomogram encompassed both HE and routine blood tests. The resource unit costs were extrapolated from the Medical Security Bureau of Guangxi Zhuang Autonomous Region website and underwent rigorous validation by the pathology department, clinical laboratory department, and the imaging center department. The unit costs for each test were obtained from the Department of Clinical Laboratory and the Department of Pathology at Guangxi Medical University Cancer Hospital in 2023, in Chinese Yuan (CNY) (1 CNY=0.14 USD).

### 2.6 Statistical analysis

**Figure 1:**
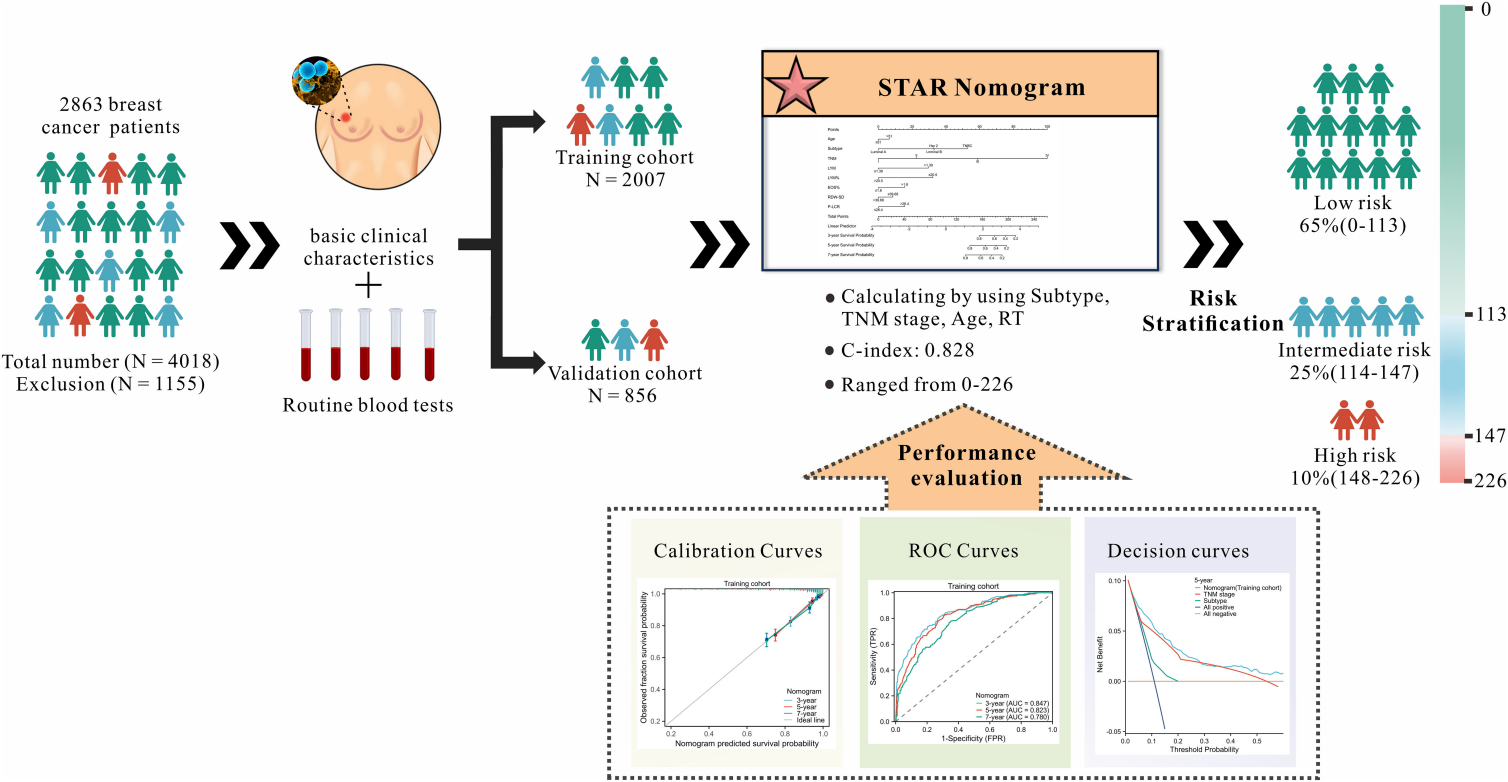
Research flowchart.

The patients in the study were randomly assigned to two groups: a training group consisting of 2007 patients and a validation group consisting of 856 patients (ratio: 7:3). The development of the nomogram was conducted using the training cohort, while the validation cohort was utilized to assess the model’s generalizability (**Figure 1**).

Statistical analyses were performed using IBM SPSS Statistical software version 23.0 and R version 4.1.3. Firstly, we performed univariate analysis on age, histologic type, grade, subtype, TNM stage, and results of 24 routine laboratory blood test items. Subsequently, significant variables (*p*<0.05) identified in the univariate analysis were subjected to multivariate Cox regression analysis. Significant variables with *p*<0.05 in the multifactorial analysis will be defined as factors with independent prognostic significance. And then using R’s rms package, a prognostic nomogram model was created using the multivariate model’s relevant factors to improve prediction. The BC patients were categorized into low, intermediate, and high-risk groups using the X-tile software. Using the Kaplan-Meier method of survival analysis, survival curves were plotted and compared using the log-rank test. The 3-, 5-, and 7-year OS nomogram was calibrated by comparing predicted survival with observed survival. Evaluate the model using calibration curves. C-index (34) and the time-dependent area under the ROC curves (AUCs) were employed to evaluate the predictive accuracy and discriminatory ability of the nomogram. When the C-index or AUC value approaches 1, it indicates excellent performance of the model. The TNM staging system was compared to the decision curve. Comparing the performance of different models at different thresholds on the same decision curve allows for a visual comparison of their performance differences. This aids in selecting the most appropriate model for decision support. All statistical tests were two-sided, and *p*<0.05 was considered statistically significant.

## 3 Results

### 3.1 Patient characteristics

We gathered data from a total of 4018 cases of BC patients. Specific exclusions(n=1155) are as follows: (I) Non-primary BC patients (n=108); (II) Ductal carcinoma *in situ* (DCIS) (n=89); (III) Primary tumor combined with other primary tumors (n=74); (IV) The patient had received any anti-tumor therapy preoperatively (n=307); (V) Inflammatory diseases, such as hematological, autoimmune or chronic/acute inflammation (132); (VI) Lost to follow-up (n=445). For the missing values generated during the construction and validation processes, we excluded them from the analysis. Only complete cases were included in our analysis. Finally, 2863 BC patients were selected for our study. 2007 patients from the training cohort and 856 patients from the validation cohort were included in the analyses. **Table 1** summarizes the demographic and clinical characteristics of patients. No differences were found between the training and validation cohorts in terms of age, Grade, Subtype, TNM stage, WBC, HGB, PLT, MONO, NEU, NEU%, LYM%, MONO%, EOSO%, EOSO, BASO%, BASO, HCT, MCV, MCH, MCHC, RDW-SD, RDW-CV, MPV, PDW, P-LCR. However, parameters including Histologic type, RBC, LYM and PCT were significantly different between the two cohorts (*p* = 0.000-0.015).

**Table 1:** Baseline characteristics of the training and validation cohorts.

Characteristic	All patients		Training cohort		Validation cohort		*P*
	No.	%	No.	%	No.	%	
**Total**	2863		2007		856		
**Age (years)**							0.928
≤51	1758	61.4%	1266	63.1%	532	62.1%	
>51	1105	38.6%	741	36.9%	324	37.9%	
**Histologic type**							0.001
IDC	2119	74.0%	1509	75.2%	610	71.2%	
ILC	76	2.7%	40	19.9%	36	4.2%	
Others	668	23.3%	458	4.9%	210	24.6%	
**Grade**							0.424
I	417	14.6%	281	14.0%	136	15.9%	
II	1270	44.4%	896	44.6%	374	43.7%	
III	1176	41.0%	830	41.4%	346	40.4%	
**Subtype**							0.513
Luminal A	405	14.1%	271	13.5%	134	15.6%	
Luminal B	1765	61.6%	1247	62.1%	518	60.5%	
Her-2	392	13.7%	276	13.8%	116	13.6%	
TNBC	301	10.6%	213	10.6%	88	10.3%	
**TNM stage**							0.939
I	645	22.5%	452	22.5%	193	22.5%	
II	1528	53.4%	1065	53.1%	463	54.1%	
III	540	18.9%	384	19.1%	156	18.2%	
IV	150	5.2%	106	5.3%	44	5.2%	
**WBC**							0.327
≤6.73	1757	61.4%	1220	60.8%	537	62.7%	
>6.73	1106	38.6%	787	39.2%	319	37.3%	
**HGB**							0.224
≤127	1375	48.0%	949	47.3%	426	49.8%	
>127	1488	52.0%	1058	52.7%	430	50.2%	
**RBC**							0.015
≤4.32	1074	37.5%	724	36.0%	350	40.9%	
>4.32	1789	62.5%	1283	64.0%	506	59.1%	
**PLT**							0.764
≤289	1754	61.3%	1226	61.1%	528	61.7%	
>289	1109	38.7%	781	38.9%	328	38.3%	
**LYM**							0.003
≤1.39	492	17.2%	317	15.8%	175	20.0%	
>1.39	2371	82.8%	1690	84.2%	681	80.0%	
**MONO**							0.271
≤0.38	1584	55.3%	1097	54.7%	487	56.9%	
>0.38	1279	44.7%	910	45.3%	369	43.1%	
**NEU**							0.773
≤4.84	2258	78.9%	1580	78.7%	678	79.2%	
>4.84	605	21.1%	427	21.3%	178	20.8%	
**NEU%**							0.486
≤55.3	832	29.1%	591	29.4%	241	28.2%	
>55.3	2031	70.9%	1416	70.6%	615	71.8%	
**LYM%**							0.139
≤20.5	288	10.1%	191	9.5%	97	11.3%	
>20.5	2575	89.9%	1816	90.5%	759	88.7%	
**MONO%**							0.872
≤4.8	590	20.6%	412	20.5%	178	20.8%	
>4.8	2273	79.4%	1595	79.5%	678	79.2%	
**EOSO%**							0.608
≤1.8	1367	47.7%	952	47.3%	415	48.5%	
>1.8	1496	52.3%	1055	52.7%	441	51.5%	
**EOSO**							0.441
≤0.16	1880	65.7%	1323	66.0%	577	67.4%	
>0.16	983	34.3%	684	34.0%	279	32.6%	
**BASO%**							0.707
≤0.8	2568	89.7%	1803	89.8%	765	89.4%	
>0.8	295	10.3%	204	10.2%	91	10.6%	
**BASO**							0.209
≤0.02	1021	35.7%	701	34.9%	320	37.4%	
>0.02	1842	64.3%	1306	65.1%	536	62.6%	
**HCT**							0.986
≤39.5	1459	51.0%	1023	51.0%	436	51.0%	
>39.5	1404	49.0%	984	49.0%	420	49.0%	
**MCV**							0.828
≤88.89	1212	42.3%	847	42.2%	365	42.6%	
>88.89	1651	57.7%	1160	57.8%	491	57.4%	
**MCH**							0.556
≤28.89	1147	40.1%	797	39.7%	350	40.9%	
>28.89	1716	59.9%	1210	60.3%	506	59.1%	
**MCHC**							0.750
≤334.79	2401	83.8%	1686	84.0%	715	83.5%	
>334.79	462	16.2%	321	16.0%	141	16.5%	
**RDW-SD**							0.723
≤39.68	1133	39.6%	790	39.3%	343	40.1%	
>39.68	1730	60.4%	1217	60.7%	513	59.9%	
**RDW-CV**							0.985
≤12.15	432	15.1%	303	15.1%	129	15.1%	
>12.15	2431	84.9%	1704	84.9%	727	84.9%	
**MPV**							0.450
≤10.01	2368	82.7%	1667	83.1%	701	81.9%	
>10.01	495	17.3%	340	16.9%	155	18.1%	
**PCT**							0.000
≤0.25	1386	48.4%	895	44.6%	491	57.4%	
>0.25	1477	51.6%	1112	55.4%	365	42.6%	
**PDW**							0.838
≤15.48	502	17.5%	350	17.4%	152	17.8%	
>15.48	2361	82.5%	1657	82.6%	704	82.2%	
**P-LCR**							0.104
≤26.4	2294	80.1%	1624	80.9%	670	78.3%	
>26.4	569	19.9%	383	19.1%	186	21.7%	

### 3.2 Univariate analysis and multivariate analysis

According to the statistical significance threshold of *p*<0.05, Age (*p* =0.005), Grade (*p* = 0.009), Subtype (*p* < 0.001), TNM stage (*p* < 0.001), and RT indicators, including WBC (*p* < 0.001), HGB (*p* = 0.005), PLT (*p* = 0.001), LYM (*p* = 0.032), MONO (*p* < 0.001), NEU (*p* < 0.001), NEU% (*p* = 0.011), LYM % (*p* < 0.001), MONO% (*p* = 0.042), EOSO% (*p* = 0.048), EOSO (*p* = 0.017), HCT (*p* = 0.006), MCV (*p* = 0.019), MCH (*p* = 0.007), MCHC (*p* = 0.012), RDW-SD (*p* = 0.010), PCT (*p* = 0.006), PDW (*p* = 0.027), P-LCR (*p* = 0.04) were associated with OS in BC patients, as determined by univariate analysis. According to the statistical significance threshold of *p*<0.05, the following factors remained independently prognostic in multivariate analysis for OS with Cox regression: age (*p* = 0.013, HR = 1.45; 95% CI: 1.08-1.95), Subtype (*p* < 0.001), TNM stage (*p* < 0.001), RT indicators LYM (*p* = 0.001, HR = 2.41; 95% CI: 1.41-4.12), LYM% (*p* = 0.004, HR = 0.46; 95%CI:0.27-0.79), EOSO% (*p* = 0.023, HR = 1.61; 95% CI: 1.61-2.42), RDW-SD (*p* = 0.042, HR = 0.73; 95% CI: 0.53-0.99), and P-LCR (*p* = 0.001, HR = 1.85; 95% CI: 1.30-2.63). **Table 2** shows the comprehensive findings of the univariate and multivariate studies.

**Table 2:** Univariate and Multivariable Analysis for overall survival of the training cohort.

Characteristic	Univariate analysis		Multivariate analysis	
	HR (95% CI)	*P*	HR (95% CI)	*P*
**Age (years) ** ≤51vs>51	1.483(1.124~1.956)	0.005	1.454(1.083~1.951)	**0.013**
**Histologic type ** IDC vs ILC IDC vs Others	1.235(0.507~3.007) 1.016(0.713~1.449)	0.897 0.643 0.929		
**Grade ** I vs II I vs III	1.211(0.746~1.966) 1.762(1.104~2.812)	0.009 0.440 0.018	1.187(0.702~2.008) 1.388(0.840~2.293)	0.339 0.522 0.201
**Subtype ** Luminal A vs Luminal B Luminal A vs Her-2 Luminal A vs TNBC	4.394(1.936~9.976) 6.717(2.839~15.890) 9.001(3.805~21.295)	<0.001 <0.001 <0.001 <0.001	3.442(1.508~7.854) 3.420(1.425~8.207) 6.318(2.636~15.144)	**<0.001** 0.003 0.006 0.000
**TNM stage ** I vs II I vs III I vs IV	2.411(1.234~4.710) 9.038(4.679~17.458) 34.020(17.332~66.77)	<0.001 0.010 <0.001 <0.001	2.056(1.043~4.055) 7.209(3.698~14.054) 27.586(13.627~55.84)	**<0.001** 0.037 **<0.001** **<0.001**
**WBC ** ≤6.73 vs >6.73	1.975(1.497~2.606)	<0.001	1.189(0.788~1.795)	0.410
**RBC ** ≤4.32 vs >4.32	0.776(0.587~1.027)	0.076		
**HGB ** ≤127 vs>127	0.673(0.510~0.888)	0.005	1.040(0.594~1.822)	0.890
**PLT ** ≤289 vs >289	1.565(1.189~2.061)	0.001	1.327(0.863~2.041)	0.197
**LYM ** ≤1.39 vs >1.39	1.656(1.044~2.628)	0.032	2.406(1.407~4.115)	**0.001**
**MONO ** ≤0.38 vs >0.38	1.933(1.457~2.565)	<0.001	1.370(0.944~1.988)	0.097
**NEU ** ≤4.84 vs>4.84	1.987(1.485~2.649)	<0.001	1.240(0.794~1.938)	0.345
**NEU% ** ≤55.3 vs >55.3	1.536(1.103~2.139)	0.011	1.373(0.948~1.989)	0.094
**LYM% ** ≤20.5 vs >20.5	0.477(0.331~0.687)	<0.001	0.464(0.274~0.785)	**0.004**
**MONO% ** ≤4.8 vs >4.8	1.495(1.015~2.204)	0.042	1.292(0.819~2.039)	0.271
**EOSO% ** ≤1.8 vs >1.8	1.327(1.003~1.755)	0.048	1.608(1.609~2.418)	**0.023**
**EOSO ** ≤**0.16 vs >0.16**	1.406(1.064~1.858)	0.017	1.061(0.717~1.569)	0.767
**BASO% ** ≤0.8 vs>0.8	0.652(0.379~1.122)	0.122		
**BASO ** <0.02 vs >0.02	1.330(0.982~1.800)	0.065		
**HCT ** ≤39.5 vs >39.5	0.674(0.509~0.893)	0.006	0.624(0.365~1.070)	0.086
**MCV ** ≤88.89 vs >88.89	0.719(0.546~0.947)	0.019	1.008(0.623~1.632)	0.973
**MCH ** ≤28.89 vs >28.89	0.687(0.512~0.904)	0.007	1.118(0.682~1.830)	0.658
**MCHC ** ≤334.79 vs >334.79	0.555(0.349~0.880)	0.012	0.750(0.450~1.251)	0.270
**RDW-SD ** ≤39.68 vs >39.68	0.695(0.527~0.916)	0.010	0.726(0.533~0.988)	**0.042**
**RDW-CV ** ≤12.15 vs >12.15	0.834(0.586~1.188)	0.315		
**MPV ** ≤10.01 vs >10.01	1.348(0.966~1.883)	0.079		
**PCT ** ≤0.25 vs >0.25	1.476(1.119~1.948)	0.006	0.851(0.560~1.294)	0.452
**PDW ** ≤15.48 vs >15.48	0.691(0.497~0.960)	0.027	1.016(0.696~1.484)	0.935
**P-LCR ** ≤26.4 vs >26.4	1.400(1.015~1.931)	0.040	1.848(1.298~2.630)	**0.001**

Bold values indicate statistically significant numerical differences.

### 3.3 Construction and validation of the nomogram

**Figure 2:**
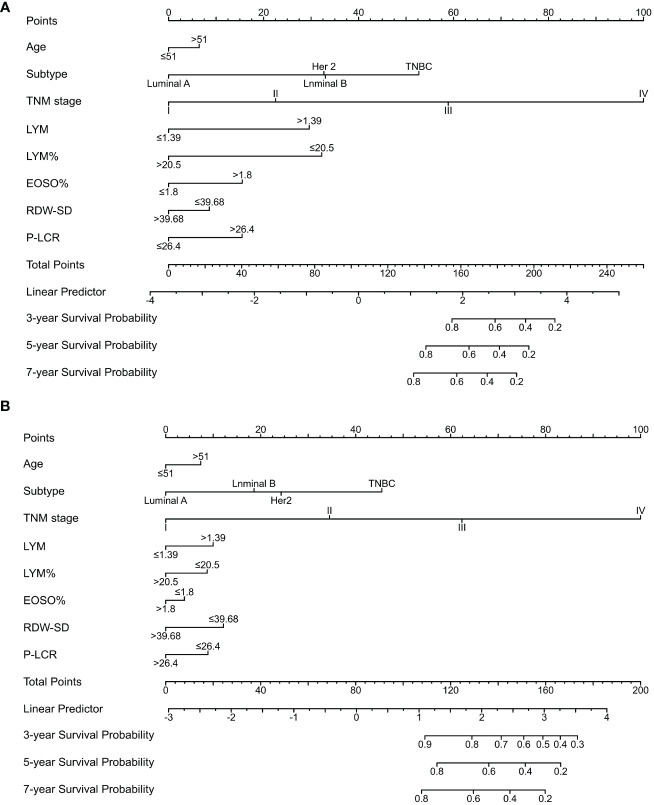
Nomogram model predicting 3-, 5- and 7- year OS in BC patients. The nomogram was used summing the points identified on the points scale for each variable. The total points projected on the bottom scales indicate the probability of 3-, 5- and 7-year survival. **(A)** The nomogram of training cohort; **(B)** The nomogram of validation cohort; LYM, lymphocyte; LYM%, percentage of lymphocyte; EOSO, Eosinophils; RDW-SD, Red blood cell distribution width - standard deviation; P-LCR, platelet-large cell ratio.

A nomogram for OS prediction, known as the Subtype–TNM stages–Age-RT indicators (STAR) was constructed based on independent prognostic factors in multivariate analysis (**Figure 2**).

**Figure 3:**
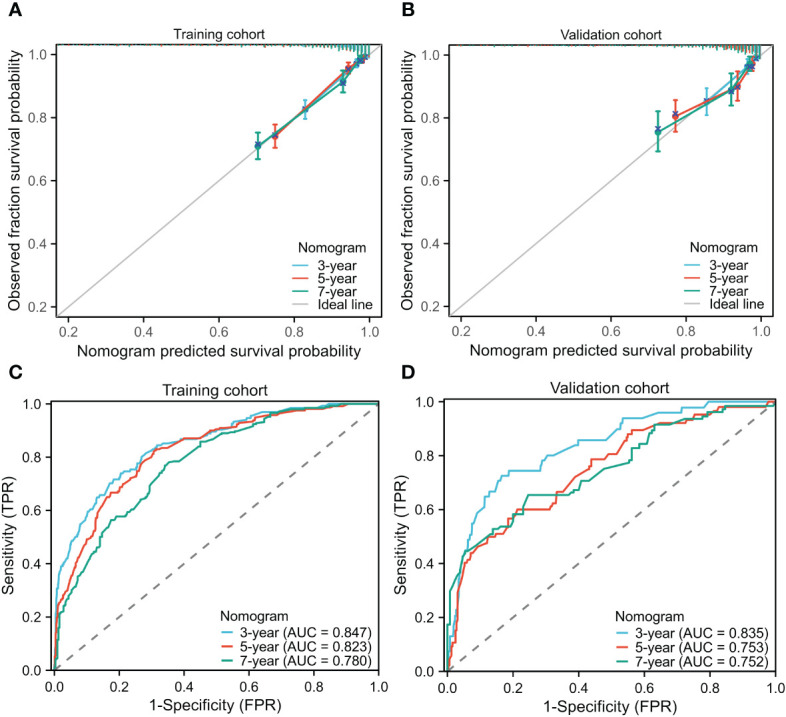
Calibration curves for predicting the 3-,5-,7-year OS in the training **(A)** and validation cohorts **(B)**. Receiver operating characteristic curves for predicting the 3-,5-,7-year OS in the training **(C)** and validation cohorts **(D)**. OS, Overall survival; AUCs, Area under curves.

The STAR prognostic nomogram model demonstrated strong accuracy in predicting the OS rate of BC patients, as indicated by a high C-index of 0.828 (95% CI, 0.813-0.843). The calibration plot, depicted in **Figure 3A**, showcased the model’s capability in accurately predicting 3-year, 5-year, and 7-year OS. In the training cohort, the AUCs of our nomogram for predicting 3-year, 5-year, and 7-year survival rates were 0.847, 0.823, and 0.780, respectively (**Figure 3C**).

In the validation cohorts, the C-index of the nomogram for predicting OS was 0.799 (95% CI, 0.773-0.825), demonstrating its strong predictive ability. The calibration curve (**Figure 3B**) displayed a favorable alignment between the 3-year, 5-year, and 7-year survival probabilities predicted by the nomogram and the actual observations. Additionally, the AUCs of our nomogram for predicting 3-year, 5-year, and 7-year survival rates in the validation group were 0.835, 0.753, and 0.752, respectively (**Figure 3D**). In both the training and validation cohorts, the nomogram model demonstrates reliable predictive accuracy and the discriminatory ability for estimating the OS of BC patients.

### 3.4 Risk stratification of OS

**Figure 4:**
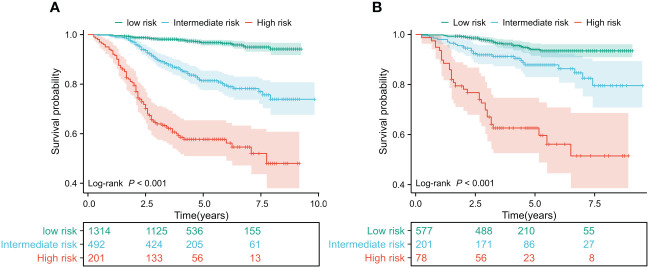
Kaplan Meier curves of predictors based on nomogram models in the training **(A)** and validation cohorts **(B)**.

In the training and validation cohorts, patients were categorized into low-risk, intermediate-risk, and high-risk groups for OS based on the X-tile (35) program’s cut-off values for the total points. In the training cohort, the patients were respectively divided into three risk groups, i.e. low-risk group (total points 0-113) accounting for 65.47%, intermediate risk group (total points 114-147) accounting for 24.51%, and high-risk group (total points 148-226) accounting for 10.01%, of which OS rates were significantly different. The OS rates for the three risk groups were 94.16%, 73.89%, and 48.01% (*p <*0.001, **Figure 4A**), respectively. Similarly, significant differences in OS were observed in the validation cohort, with OS rates of 93.45% for the low-risk group, 79.54% for the intermediate-risk group, and 51.46% for the high-risk group (*p* < 0.001, **Figure 4B**). Patients in the low-risk group have a better prognosis and may require fewer treatment interventions and monitoring measures. Conversely, patients in the high-risk group may require more intensive interventions and close monitoring. This information is useful for physicians in developing personalized treatment plans and subsequent management strategies for patients. This STAR nomogram has great potential in individual prognosis prediction and enables approximately 90% of BC patients to undergo less intensive surveillance leading to a reduction in the health burden.

### 3.5 Comparison among the STAR nomogram, TNM staging system and nomograms based on molecular profiling and imaging features

We compared the STAR nomogram with the TNM staging system and nomograms based on molecular profiling and imaging features. Through a meticulous examination of the PubMed and Web of Science databases, utilizing the specified search terms mentioned above, we initially identified 1,013 pieces of literature. Upon screening abstracts and full-texts, we found that 292 of the literature pieces were limited to specific breast cancer subtypes, 329 investigations lacked the association between BC survival and molecular profiling or imaging features, and 36 studies lacked both a training cohort and a validation cohort with a C-index value. Additionally, 168 literature pieces were unrelated to breast cancer. Out of these, 146 literature pieces were related to bioinformatics research, and 40 literature pieces were not research articles. Ultimately, the studies by Silei Sui et al. and Xiaojun Xu et al. were meticulously chosen to represent nomograms based on molecular profiling and imaging features, respectively. In the training cohort, the STAR nomogram exhibited a C-index of 0.828 (95% CI, 0.813-0.843), signifying its superior predictive accuracy in contrast to the TNM staging system (C-index 0.766, 95% CI, 0.749-0.784) and the molecular profiling and imaging features system (C-index 0.665, 95% CI, 0.653-0.677) (**Table 3**). Similarly, in the validation cohort, the STAR nomogram outperformed other prediction models. It achieved a C-index of 0.847, whereas the C-index values for TNM staging, molecular profiling, and imaging features systems were 0.756, 0.691, and 0.758, respectively. These findings reinforce the superior predictive capability of the STAR nomogram over traditional TNM staging, molecular profiling, and imaging features systems in the validation cohort.

**Table 3:** The C-index values of different prediction models.

Factors	Training cohort	Validation cohort	Unit price (USD)
	C-index (95% CI)	C-index (95% CI)	
**STAR Nomogram** **TNM Stage** **Molecular profiling** **Imaging features**	0.828 (0.813-0.843) 0.766 (0.749-0.784) 0.665 (0.653-0.677) 0.845 (0.793-0.912)	0.799 (0.773-0.825) 0.756 (0.731-0.782) 0.691 (0.663-0.719) 0.758 (0.723-0.801)	$26.3 $22.4 $862.4 $722.4

Furthermore, **Table 3** presents a comparative analysis of the relative costs per BC patient associated with each prediction strategy. The current standard of care, TNM staging, had an expected cost of $22.4. In contrast, the STAR nomogram demonstrated superior cost-effectiveness, with a cost of $26.3, significantly lower than molecular profiling ($862.4) and Imaging features ($722.4). We believe that the STAR nomogram stands out as a more cost-effective option among these prediction models, given its excellent prognostic performance.

**Figure 5:**
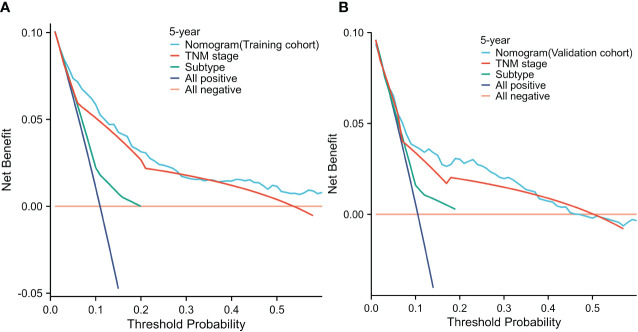
Decision curve analysis for 5-year survival predictions in the training **(A)** and validation cohorts **(B)**.

In both the training cohort (**Figure 5A**) and the validation cohort (**Figure 5B**), the decision curve analysis (The threshold probability:0-0.6) revealed that the nomogram provided greater clinical benefit across a wider range of threshold probabilities for predicting OS. These findings indicate that the STAR nomogram model outperformed the TNM staging system in terms of clinical utility and net benefit in predicting OS. The decision curve analysis further supports the superiority of the STAR nomogram model as a valuable tool for risk prediction and clinical decision-making in BC patients.

## 4 Discussion

In this study, we embarked on a systematic evaluation of the prognostic significance of routine blood test (RT) parameters in predicting the outcomes of breast cancer patients. To our knowledge, this marks the pioneering effort to comprehensively assess the prognostic value of RT parameters in BC. Furthermore, through the integration of fundamental clinical characteristics with RT parameters, we successfully developed a predictive nomogram model, termed the STAR nomogram. This model serves as an effective tool for forecasting overall survival in BC patients, providing invaluable prognostic insights. Remarkably, in comparison to the conventional TNM staging, molecular profiling and imaging feature systems, the STAR nomogram exhibits exceptional predictive performance.

Univariate and multivariate Cox regression analysis of RT parameters in this study showed that lymphocyte count, lymphocyte ratio, percentage of eosinophils, red blood cell distribution width, and platelet large cell ratio had independent prognostic values. Because of its advantages of easy access and low price, RT parameters play an increasingly important role in the prediction of cancer (36, 37). Consistent with this study, many studies have also shown that the number of peripheral blood lymphocytes has good prognostic value. Anosheh Afghahi et al. found that low lymphocyte count (LC) was closely related to poor OS of BC (38). The retrospective analysis of Sung Min Ko et al. showed that patients with high LC had better disease-free survival (DFS) than patients with low LC (26). Lymphocytes are a kind of inflammatory cells, which play an important role in the development of breast cancer (39, 40). Peripheral lymphocytes can migrate to the tumor site and infiltrate the tumor microenvironment (41, 42). CD8^+^ cytotoxic T lymphocytes increase antitumor immunity, however, exhausted CD8^+^ T lymphocytes and regulatory T cells suppress antitumor immunity (43, 44). Therefore, further research is needed on the mechanism of peripheral lymphocytes affecting tumors. Eosinophils are the primitive cells of the innate immune system, which have a powerful ability to influence local immunity and tissue remodeling during homeostasis and disease (45, 46). In our study, eosinophils were an independent prognostic factor of BC, which was also confirmed by the study of Concetta Elisa Onesti et al. (27). There is controversy about the tumor effect of eosinophils, which have direct or indirect anti-tumor activity, but sometimes also promote the development of tumors (47–49). Therefore, the exact mechanism by which eosinophils play a role in BC needs further study. Red blood cell distribution width reflects the degree of heterogeneity of red blood cell volume (50). As a laboratory hematological parameter, it can also be used as an independent prognostic marker to predict the survival of cancer patients (51–54). There are few studies on P-LCR in cancer (55). Platelets serve a crucial role in cancer progression and inflammation, according to new evidence (56, 57). At present, no study has explored the prognostic value of platelet large cell ratio in BC. Our study shows for the first time that the platelet large cell ratio is an independent prognostic factor for BC. Platelet large cell ratio (P-LCR), is defined as the proportion of platelets larger than 12 fL (58). The large platelet ratio is mainly used to show the morphology of platelets and is a good monitoring tool for platelet activity. Large platelets are more likely to bind more fibrinogen on their surface and have higher protein phosphorylation levels after thrombin stimulation (59). Tumor cells are capable of inducing a real platelet aggregation, largely mediated by fibrinogen binding to integrin αIIbβ3 and reinforced by fibrin formation (60). The extent of platelet activation influences several effector factors, such as Vascular Endothelial Growth Factor (VEGF), Platelet-derived growth factor (PDGF), and transforming growth factor-β (TGF-β), which influence vascular maturation in the tumor microenvironment and mediate cancer cell invasion (61), which are associated with the survival rate of BC patients. However, the mechanism of platelet number and size changes in BC is unclear and needs further study.

Our STAR nomogram model represents a significant improvement in the accuracy of prognostic predictions for BC patients. Compared with the existing staging system, the model has higher prediction accuracy and discrimination ability. With the development of medicine and informatics, nomograms can generate the personal digital probability of clinical events, which meets our demand for biological and clinical integrated models, leading to the ubiquitous appearance of nomograms on the internet and in medical journals (62–64). Several other predictive models have been established for BC patients. For instance, Yufen Zheng et al. established a prediction model based on preoperative fibrinogen albumin ratio and platelet lymphocyte ratio (FAR-PLR score) to predict the prognosis of BC patients (65). Fei Lin et al. constructed a nomogram based on the nutritional risk index (NRI) and clinical characteristics (66). There are also nomograms based on imaging histology (67), transcriptomics data (68), etc. that can also make a good prediction for BC patients. Our STAR model achieved a C-index of 0.828 (95% CI, 0.813-0.843), surpassing the average C-index of 0.74 (69) and other similar nomograms (the C-index for the nomograms based on FAR-PLR score and the nutritional risk index were 0.652 and 0.793, respectively). Notably, the acquisition of RT parameters is non-invasive, cost-effective, and easily obtainable. Importantly, it is highly adaptable and can be widely applied, even in primary healthcare settings. Moreover, the STAR nomogram offers the fastest potential turnaround time, ranging from just 0.5 to 6 hours, as opposed to the molecular profiling and imaging features system, which can take up to 10 days to yield results. The prediction model based on RT parameters presented in this study not only enhances the economic benefits for patients but also aids clinicians in making informed treatment decisions. Additionally, it contributes to the advancement of personalized medicine. By integrating multiple predictive factors, the STAR nomogram can estimate a patient’s survival probability. Using the Nomogram, the total score for each patient can be calculated based on assigned scores for each risk variable, with higher scores being associated with an adverse prognosis. These predictive results provide patients and clinicians with a more specific and objective understanding of treatment efficacy and prognosis. Patients can gain insight into their own risks and potential treatment outcomes, enabling them to make more informed decisions. Meanwhile, clinicians can better engage in individualized follow-up and subsequent treatment planning.

Although our nomogram provided clinicians with a useful tool for selecting and planning treatment strategies for patients with BC, our study has several limitations. First, there could be potential selection bias inherent in any retrospective study, as patient data were collected from medical records. This could have influenced the representativeness of our study population and the generalizability of our findings. Second, our study solely examined BC patients’ OS prognostic values and did not assess our nomogram’s DFS prediction ability. DFS can help assess the efficacy of treatment in controlling tumor recurrence and progression (70). It was a more effective clinical implementation of the nomogram when OS and DFS were included. In subsequent studies, we will continue to collect DFS data and assess the model’s ability to predict DFS. Third, the nomogram predictions lack of external validation cohorts, and we will collect more external data in the follow-up study to verify this model. In subsequent studies, we plan to collaborate with other institutions or international partners to share data or collect additional samples. This will increase the sample size and enhance the reliability and statistical power of validation. We also intend to perform multicenter data validation, cross-validation, and internal validation to repeatedly optimize and update the model, reducing potential influences on model robustness while improving its reliability and generalizability.

## 5 Conclusion

Overall, our study confirms that RT parameters can be used as a promising prognostic factor for BC patients. The STAR nomogram which is facilitates accurate, cheap, reliable and simple-to-use prediction for predicting OS of BC patients. It is entirely objective, being based on 4 of the most common clinical parameters. Thus, this STAR nomogram will be a useful tool for clinicians’ decision-making and individual patient consultation. In future work, whether active intervention guided by prognostic laboratory markers will improve the prognosis assessment of patients needs further investigation.

## Data availability statement

The datasets generated and/or analyzed during the current study are not publicly available due to that the data also forms part of other ongoing studies but are available from the corresponding author on reasonable request.

## Ethics statement

The studies involving humans were approved by Guangxi Medical University Cancer Hospital Ethical Review Committee. The studies were conducted in accordance with the local legislation and institutional requirements. The participants provided their written informed consent to participate in this study.

## Author contributions

CW: Writing – review & editing. YL: Writing – original draft, Data curation, Formal analysis. DM: Writing – original draft, Data curation, Formal analysis, Software. QL: Writing – original draft, Validation. ZL: Data curation, Writing – original draft. ML: Data curation, Writing – original draft. YQ: Data curation, Investigation, Methodology, Software, Writing – original draft. MF: Writing – review & editing, Conceptualization, Funding acquisition.
